# A single dose of dezocine suppresses emergence agitation in preschool children anesthetized with sevoflurane-remifentanil

**DOI:** 10.1186/s12871-017-0446-8

**Published:** 2017-11-22

**Authors:** Li-Jun An, Yang Zhang, Zheng Su, Xian-Long Zhang, Hai-Lin Liu, Zhi-Jie Zhang, Jian-Lin Hu, Shi-Tong Li

**Affiliations:** 1 Department of Anesthesiology, Shanghai General Hospital of Nanjing Medical University, University, No. 100 Haining Road, Shanghai, 200030 China; 2 Department of Anesthesiology, Huai’an First People’s Hospital of Nanjing Medical University, Huai’an, Jiangsu China

**Keywords:** Dezocine, Emergence agitation, Preschool children, Sevoflurane-remifentanil

## Abstract

**Background:**

Emergence agitation (EA) is a common phenomenon in preschool children during emergence from general anesthesia. This study evaluated the safety and efficacy of dezocine for emergence agitation in preschool children anesthetized with sevoflurane-remifentanil.

**Methods:**

A total of 100 preschool children, scheduled for elective laparoscopic repair of an inguinal hernia by high ligation of the hernia sac under sevoflurane-remifentanil anesthesia were randomized into two groups: Group C (*n* = 50) received Ringer’s lactate 10 mL and Group D received Ringer’s lactate 10 mL containing dezocine 0.1 mg/kg, postoperatively.

**Results:**

Incidence of EA, defined as a score ≥ 3 on Aono’s four point scale or Pediatric Anesthesia Emergence Delirium (PAED) score ≥ 10 in the PACU (10% vs. 76%) and the percentage of patients with severe EA (PAED score ≥ 13) (12% vs. 76%) were significantly lower in Group D compared to Group C (*P* < 0.05). Mean Children and Infants Postoperative Pain Scale (CHIPPS) score was significantly lower in Group D compared to Group C (1.2 ± 0.5 vs. 5.2 ± 0.6; P < 0.05). Patients need for fentanyl (18% vs. 4%) or propofol rescue (20% vs. 0) was significantly greater in Group C compared to Group D. No significant differences in other relative aspects after surgery between groups.

**Conclusion:**

Administration of dezocine 0.1 mg/kg decreased the incidence and severity of EA in preschool children that had undergone laparoscopic repair of an inguinal hernia by high ligation of the hernia sac under sevoflurane-remifentanil anesthesia.

**Trial registration:**

A single dose of dezocine suppresses emergence agitation in preschool children anesthetized with sevoflurane-remifentanil effectively: A double-blind, prospective, randomized, controlled study, Chinese Clinical Trial Registry (ID: ChiCTR-IOR-16010033), retrospectively registered on November 21, 2016.

## Background

Laparoscopic repair of an inguinal hernia by high ligation of the hernia sac is a common minimally invasive pediatric surgical procedure. Sevoflurane, combined with low-dose remifentanil, is an anesthetic procedure that has been widely used in hernia surgery. Although sevoflurane-remifentanil anesthesia is associated with a smooth onset, rapid induction, prompt emergence, and fast recovery, it can result in emergence agitation (EA) [1]. EA is defined as psychomotor agitation during emergence from general anesthesia, and is a frequent problem among pediatric patients [2]. Indeed, the incidence of EA has been estimated at 10–66% in pediatric patients following sevoflurane-remifentanil anesthesia [3].

EA can result in physical harm to the child or to surgical staff. Although self-limiting, EA is disturbing for both parents and care-givers [4]. Evidence suggests that causative factors of EA in preschool-aged children include rapid emergence, psychological and neurological immaturity, sevoflurane-induced noradrenaline release in the brain, pain, and anxiety [5].

As pain and anxiety are implicated in the etiology of EA, analgesic and sedative agents such as midazolam, α2 adrenergic receptor agonists such as propofol, and opioids have been used to minimize EA [6–10]. These therapies have various degrees of success, as well as their own respective side effects when treating EA [11].

Dezocine is an analgesic and sedative that is used for perioperative pain management. We hypothesized that postoperative dezocine administration may decrease the incidence and severity of EA in pediatric patients. Dezocine is a full agonist of the κ-receptor and partial agonist of the μ-receptor without μ-receptor dependence. The analgesic effect of dezocine is approximately equipotent with morphine. It has a minor ceiling effect for respiratory depression accompanying its analgesic activity, and its sedative effect is well tolerated in a single dose [12]. The objective of this study was to investigate the safety and efficacy of postoperative dezocine administration on the incidence and severity of EA in preschool children undergoing laparoscopic repair of an inguinal hernia by high ligation of the hernia sac under sevoflurane-remifentanil anesthesia.

## Methods

### Study population and study design

Preschool children undergoing laparoscopic repair of an inguinal hernia by high ligation of the hernia sac at our institution between December 2014 and November 2016 were eligible for this study. Inclusion criteria were 1) American Society of Anesthesiologists (ASA) score I or II; and 2) scheduled for elective laparoscopic repair of an inguinal hernia by high ligation of the hernia sac under sevoflurane-remifentanil anesthesia. Exclusion criteria were 1) psychiatric or neurological disease; 2) sleep apnea; 3) treatment with sedative or opioid two weeks prior to surgery and 4) body mass index (BMI) ≥40 kg/m^2^. Study participants were randomly allocated into Group C (control group, *n* = 50) and Group D (dezocine group, n = 50) using a computer generated table of random numbers.

This study was approved by the Ethics Committee of Huai’an First People’s Hospital, affiliated with Nanjing Medical University, China (Approval # HA141001S). Written informed consent was obtained from the patients’ parents or legal guardians.

Children were fasted for ≥4 h before induction of anesthesia and received no premedication. Upon arrival in the operating room, heart rate (HR), pulse oximetry (SpO2) and non-invasive blood pressure (BP) monitoring were applied. One experienced anesthesiologist administered anesthesia, performed tracheal intubation, and assessed intubation. Anesthesia was induced with 8% sevoflurane in oxygen flowing at 6 L/min through a pediatric anesthesia breathing circuit with a 1.5 L re-breathable bag. Initially, spontaneous respiration was assisted; subsequently, respiration was manually controlled. An intravenous cannula was sited after disappearance of the eyelash reflex. Dexamethasone 0.2 mg/kg and ondansetron 0.15 mg/kg were administered through the cannula. The concentration of sevoflurane was decreased to 1.3 minimum alveolar concentration (MAC). Tracheal intubation was attempted using a tracheal tube (size formula: internal diameter = 4 + yr./4), the muscle relaxant atracurium 1.0 mg/kg, and fentanyl 1 μg/kg. Anesthesia was maintained with 0.8–1.2 MAC sevoflurane in combination with remifentanil (0.5 μg/kg/h), BP and HR within 20% of the baseline value, and bispectral index (BIS) values between 40 and 60. Ventilation was controlled to maintain end tidal carbon dioxide (ETCO2) levels between 35 and 40 mmHg and oxygen flow at 2 L/min.

Skilled surgeons performed the operations. All incisions were infiltrated with 0.25% ropivacaine before surgery. At the completion of the surgery, remifentanil was discontinued and fentanyl 1 μg/kg was administered. Atropine 0.01 mg/kg and neostigmine 0.04 mg/kg were used to reverse residual neuromuscular blockade. Patients in Group C received 10 mL Ringer’s lactate; patients in Group D received dezocine 0.1 mg/kg (Yangzi River Co., Jiangsu, China) in 10 mL Ringer’s lactate. Concurrently, sevoflurane was terminated and controlled ventilation with 8 L/min of oxygen was instituted. Extubation was performed when the cough reflex and regular respiration were regained. After extubation, subjects were transferred to the post-anesthesia care unit (PACU). Oxygen (6 L/min) was administrated via a face-mask in the PACU.

### Clinical evaluation

Primary outcomes of the study were the incidence and severity of EA in the PACU. Secondary outcomes of the study were time to extubation, emergence time, Children and Infants Postoperative Pain Scale (CHIPPS) score, incidence of rescue drug administration, time in the PACU, and Ramsay sedation score.

Emergence time after extubation was defined as time to eye opening or purposeful movement in the PACU. Consciousness after emergence was evaluated using the Ramsay sedation scale [13]. In the PACU, a study investigator blinded to the study groups simultaneously assessed the incidence of postoperative EA defined as a score ≥ 3 on Aono’s four point scale or Pediatric Anesthesia Emergence Delirium (PAED) PAED score ≥ 10, and the severity of postoperative EA was described by the distribution of EA scores on the PAEDscale [1, 14]; a score ≥ 13 was classified as severe EA and a score ≥ 10 - <13 was classified as moderate EA. Postoperative pain was assessed by the CHIPPS [15]. A bolus of fentanyl 1 μg/kg was injected as a rescue medication for severe EA or a CHIPPS pain score > 5. Propofol 1 mg/kg was administered if EA was not attenuated within 5 min. Patients were discharged from the PACU to the ward when they recovered from anesthesia, defined as an Aldrete score < 9 [16].

### Statistical analysis

Statistical analyses were conducted using IBM SPSS Statistics for Windows Version 20.0 (IBM Corp., Armonk, NY, USA). Based on a previous study in children undergoing cleft lip surgery under sevoflurane anesthesia that reported a 25% decrease in the incidence of EA as clinically significant [17], the sample size for the current study was calculated as 50 patients in each group (alpha value, 0.05; beta-value, 0.80).

Continuous variables were analyzed with Student’s *t*-test. Categorical variables including gender, ASA, and incidence of EA are reported as frequency, percentage, and risk ratios (RRs) with corresponding 95% confidence interval (CIs) and were compared using chi-square test and correction or Fisher’s exact probability test. A *P* value less than 0.05 was considered statistically significant.

## Results

### Study sample

**Fig. 1:**
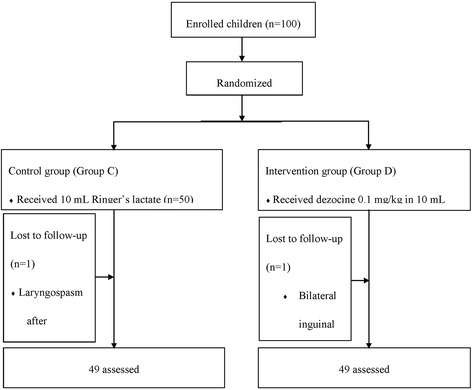
CONSORT diagram. A total of 100 children were enrolled the study and allocated into two equal groups. Two children were lost to follow up, one because of bilateral inguinal hernia, the other due to laryngospasm after extubation and the use of muscle relaxant

This study included a consecutive sample of 100 pediatric patients (55 male/23 female; mean age 4.6 ± 1.9 years, range 1–6 years) (Fig. 1). All surgeries were successful. Two cases were lost to follow-up, one in Group C that experienced serious laryngospasm after extubation requiring the use of a muscle relaxant and one in Group D who suffered from bilateral inguinal hernia. The final analysis included 49 patients in Group C (29 male/20 female; mean age 4.9 ± 1.6 years, range 1–6 years) and 49 patients in Group D (26 male/23 female, mean age 4. 2 ± 2.1 years, range, 1–6 years). Patients’ baseline demographic and clinical characteristics are shown in Table 1. There were no significant differences between groups.

**Table 1:** Patients baseline demographic and clinical characteristics

	Group C (n = 49)	Group D (n = 49)	*p*-value
Age (yr)	4.9 ± 1.6	4.2 ± 2.1	0.067
Sex (M/F)	29/20	26/23	0.541
Weight (kg)	18.4 ± 3.1	17.5 ± 4.3	0.238
Height (cm)	78.3 ± 10.2	80.5 ± 12.3	0.338
BMI	19.5 ± 3.2	20.1 ± 2.8	0.326
ASA (I/II)	46/3	46/3	1.000
Duration of surgery (min)	35.1 ± 6.9	36.8 ± 8.0	0.263

Data are presented as numbers or mean ± SD. Group C = Ringer’s lactate, Group D = dezocine 0.1 mg/kg. M: male, F: female. BMI: Body Mass Index; ASA: American Society of Anesthesiologist

### Outcomes

**Fig. 2:**
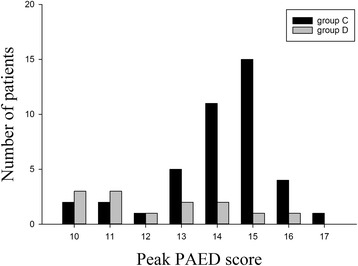
Distribution of PAED scores

Primary and secondary outcome data are shown in Table 2 and Fig. 2. Incidence of EA in the PACU (10% vs. 76% and 27% vs. 86%, *P* < 0.05), the percentage of patients with severe EA (88% vs. 46%, *P* < 0.05), and the percentage of patients with moderate EA (12% vs. 54%, *P* < 0.05) were significantly lower in Group D compared to Group C. There were no significant differences in mean time to extubation and mean emergence time between groups. Mean CHIPPS score was significantly lower in Group D compared to Group C (1.2 ± 0.5 vs. 5.2 ± 0.6; *P < 0.05*). Patients need for fentanyl (18% vs. 4%) or propofol rescue (20% vs. 0%) was significantly greater in Group C compared to Group D. There was no significant difference in mean time to discharge from the PACU between groups. There were no significant differences in the incidence of postoperative events including desaturation, headache, nausea, and vomiting between the two groups.

**Table 2:** Emergence agitation and recovery profiles

	Group C (n = 49)	Group D (*n* = 49)	p-value
Time to extubation (min)	8.1 ± 1.6	8.6 ± 1.4	0.103
Time from extubation to emergence (min)	13.5 ± 2.1	14.1 ± 1.7	0.123
Discharged from PACU (min)	46.4 ± 8.7	36.5 ± 9.3	4.032
Incidence of EA by PAED scale score ≥ 10 (%)	42 (86%)	13 (27%)	<0.0001
PAED score ≥ 10 (moderate EA)	5 (12%)	7 (54%)	<0.0001
PAED score ≥ 13 (severe EA)	37 (88%)	6 (45%)	<0.0001
CHIPPS pain score	5.2 ± 0.6	1.2 ± 0.5	<0.0001
EA rescued by fentanyl	9 (18%)	2 (4%)	0.025
EA rescued by propofol	10 (20%)	0 (0%)	0.001
Ramsay sedation score	1.2 ± 0.5	1.9 ± 0.4	1.533
Desaturation	0	0	
Nausea	0	0	
Vomiting	0	0	
Headache	0	0	

Data are presented as mean ± SD, percentages. *EA* Emergent agitation, *PAED* Pediatric Anesthesia Emergence Delirium, *PACU* Post-anesthetic care unit

**Fig. 3:**
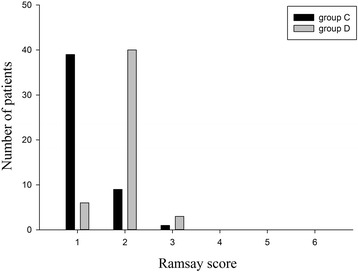
Ramsey sedation scores for individual patients. The registration information can be found on the following website: http://www.chictr.org.cn/searchproj.aspx

There was no significant difference in mean Ramsey sedation score between groups. However, more patients in Group C were evaluated as level 1, which indicates they were anxious, agitated or restless, compared to Group D, while more patients in Group D were evaluated as level 2 (cooperative, orientated, tranquil) or level 3 (responsive to commands only) compared to Group C (Fig. 3).

## Discussion

This study demonstrated that a single dose of dezocine 0.1 mg/kg reduced the incidence and severity of postoperative EA in preschool children undergoing laparoscopic repair of an inguinal hernia by high ligation of the hernia sac under sevoflurane-remifentanil anesthesia. Furthermore, dezocine reduced the use of fentanyl and propofol rescue therapy during EA. Although there was a small increase in emergence time in the patients treated with dezocine, there was no delay in discharge from the PACU and no significant differences in the incidence of postoperative oxygen desaturation, headache, nausea, and vomiting between the dezocine and control groups, which suggests that dezocine is not associated with any adverse events in the patient population analyzed.

In our study, dezocine reduced the incidence of EA to 10% compared to 76% in the control group. These data are in accordance with a previous study comparing the effects of dezocine, fentanyl, or placebo on the incidence and severity of EA in children undergoing adenotonsillectomy under sevoflurane anesthesia. Findings showed that the incidence (PAED score ≥ 10) and severity of EA (Severe: Aono’s score ≥ 3) was comparable following a single dose of dezocine 0.1 mg/kg (incidence, 32%; severe 24%) and fentanyl 1 μg/kg (incidence, 34%; severe 26%), but lower than placebo (incidence, 61%; severe 50%) [18].

The current study used the PAED scale to assess the severity of EA. The PAED scale is a valid and reliable measure of EA in children; however, there is no consensus on the threshold value that indicates the presence of EA. Previous studies have used PAED threshold values ranging from 10 to 16 to identify EA [19–21]. Bajwa et al. reported that a PAED score > 12 provides greater sensitivity and specificity than a PAED score ≥ 10; therefore, we defined moderate EA as ≥10 and <13, and severe EA as ≥13 [22].

In the current study, dezocine reduced the use of fentanyl and propofol rescue therapy during EA. Many factors contribute to EA, including patient age, type of surgery, anesthetic procedure, and rapid emergence after surgery. Importantly, pain has been identified as a significant factor in EA. The analgesic effect of dezocine was reflected by a significantly lower CHIPPS score in patients in Group D compared to Group C. Previous studies showed that fentanyl effectively prevents EA in children following sevoflurane anesthesia [23]. However fentanyl also results in chest-wall rigidity and respiratory depression. In the current study, patients in both groups with severe EA were administered intravenous fentanyl. Significantly more patients in Group C required fentanyl rescue compared to Group D, which suggests that dezocine may enhance the analgesic effect of fentanyl. In the patients in our study, pain was mainly caused by surgical incision, stimulation of the gut, and inflammation. Incisional pain was blocked by ropivacaine. Visceralgia was relieved by the κ-receptor agonist effect of dezocine in Group D.

The Ramsay sedation score is the most widely used clinical measure of sedation. In this study, patients were evaluated upon admission to the hospital by two neurologists who were not involved in the treatment procedure. The Ramsay sedation score was within the normal range for each child. Postoperatively, there was no significant difference in Ramsay sedation scores between Group C and Group D; however, the number of patients with different levels of sedation varied between groups. A higher number of patients in Group C were evaluated as anxious, agitated or restless compared to Group D, while a greater number of patients in Group D were evaluated as cooperative, orientated, and tranquil or responsive to commands only, compared to Group C. These data indicate that dezocine may reduce the severity of EA though a sedative effect. Evidence suggests that the analgesic and sedative effect of dezocine may be due to inhibition of norepinephrine and serotonin reuptake by their respective transporter proteins. Indeed, in a rat model of chronic constriction injury, dezocine inhibited norepinephrine and serotonin reuptake in a dose dependent manner [24].

This study was associated with several limitations. Firstly, it was a single center study. Multicenter studies are required to verify the safety and efficacy of dezocine for EA in pediatric patients. Secondly, distinguishing between postoperative pain and EA is challenging. Our data may have been confounded by the analgesic effect of dezocine on postoperative pain; indeed, mean CHIPPS score was significantly lower in Group D compared to Group C. Lastly, there was no significant difference in mean Ramsey sedation score between groups, which was unexpected, as dezocine is thought to have sedative effects. Further studies with larger sample sizes are required to verify our findings and elucidate the mechanism of action of dezocine on EA.

## Conclusions

In conclusion, postoperative administration of dezocine 0.1 mg/kg decreased the incidence and severity of EA in the PACU in preschool children that had undergone laparoscopic repair of an inguinal hernia by high ligation of the hernia sac under sevoflurane-remifentanil anesthesia.

## Abbreviations

ASA: American Society of Anesthesiologists
BIS: bispectral index
BMI: body mass index
BP: non-invasive blood pressure
CHIPPS: Children and Infants Postoperative Pain Scale
CI: confidence interval
EA: emergence agitation
HR: heart rate
MAC: minimum alveolar concentration
PACU: post-anesthesia care unit
PAED: Pediatric Anesthesia Emergence Delirium
RRs: risk ratios
